# GHS-R in brown fat potentiates differential thermogenic responses under metabolic and thermal stresses

**DOI:** 10.1371/journal.pone.0249420

**Published:** 2021-04-01

**Authors:** Jong Han Lee, Ligen Lin, Xiangcang Ye, Christian Wolfrum, Yingjie Chen, Shaodong Guo, Yuxiang Sun

**Affiliations:** 1 USDA/ARS Children’s Nutrition Research Center, Department of Pediatrics, Baylor College of Medicine, Houston, TX, United States of America; 2 Department of Nutrition, Texas A&M University, College Station, TX, United States of America; 3 Department of Health Science and Technology, ETH Zurich, Schwerzenbach, Switzerland; 4 Department of Physiology and Biophysics, University of Mississippi Medical Center, Jackson, MS, United States of America; University of Texas Health Science Center at Houston, UNITED STATES

## Abstract

In response to cold or diet, fatty acids are dissipated into heat through uncoupling protein 1 (UCP1) in brown adipose tissue (BAT). This process is termed non-shivering thermogenesis, which is important for body temperature maintenance and contributes to obesity pathogenesis. Thermogenic enhancement has been considered a promising anti-obesity strategy. Ghrelin and its receptor Growth Hormone Secretagogue Receptor (GHS-R) have critical roles in energy intake, nutrient sensing, and lipid metabolism. We previously reported that global *Ghsr*-knockout mice have increased energy expenditure due to enhanced thermogenesis. To determine the site of action for GHS-R mediated thermogenesis, we generated brown adipocyte-specific *Ghsr* knockout mice (*UCP1*-Cre^ER^/*Ghsr*^*f/f*^) and assessed thermogenic responses under regular diet (RD) fed homeostatic metabolic state or high-fat diet (HFD) fed metabolically-impaired obese state, under normal or cold housing environment. Under a RD-feeding, *UCP1*-Cre^ER^/*Ghsr*^*f/f*^ mice showed increased body fat and a slightly elevated core body temperature under cold but not under normal temperature. Consistently, the expression of thermogenic genes in BAT of RD-fed *UCP1*-Cre^ER^/*Ghsr*^*f/f*^ mice was increased in reposes to cold. Under HFD feeding, HFD-fed *UCP1*-Cre^ER^/*Ghsr*^*f/f*^ mice showed no difference in body fat or body temperature under either normal or cold exposure. Interestingly, the expression of thermogenic genes in BAT of HFD-fed *UCP1*-Cre^ER^/*Ghsr*^*f/f*^ mice was upregulated under normal temperature but downregulated under cold exposure. Overall, our data show that GHS-R has cell-autonomous effect in brown adipocytes, and GHS-R regulates BAT thermogenic activity in a temperature- and metabolic state-dependent manner. The thermogenic effect of GHS-R in BAT is more pronounced in cold environment and differentially variable based on metabolic state; under cold exposure, GHS-R inhibition in BAT activates thermogenesis under homeostatic state but suppresses thermogenesis under obese state. Our finding collectively suggests that GHS-R in BAT, acting as a “metabolic thermostat”, differentially regulates thermogenesis in response to different metabolic and thermal stimuli.

## Introduction

Brown adipose tissue (BAT) is a key site of non-shivering thermogenesis, converting food-generated fatty acids into heat [1, 2]. This energy dissipating aspect of BAT is considered favorable for metabolism as it increases energy expenditure and reduces fat deposition in the body. Uncoupling protein 1 (UCP1), an inner mitochondrial membrane protein, is essential to non-shivering thermogenesis in brown adipocytes [3]. UCP1 uncouples ADP phosphorylation in mitochondrial respiration, which is a critical process of heat production in the mitochondria [4]. BAT is involved in diet-induced thermogenesis, as well as cold-induced thermogenesis, that helps to maintain body temperature in a cold environment [1, 5, 6]. Regulators involved in energy homeostasis have been considered as plausible targets for the treatment of obesity and diabetes [7–9]. The energy consumption property of BAT is being recognized as a beneficial attribute in maintaining metabolic homeostasis and a potential therapeutic strategy for obesity.

Ghrelin is an orexigenic hormone involved in the regulation of nutrient-sensing, meal initiation, and appetite stimulation [10, 11]. Ghrelin and its receptor Growth Hormone Secretagogue Receptor (GHS-R), have been implicated in the pathogenesis of obesity and type 2 diabetes [12]. Our previous studies have shown that global ablation of GHS-R increases energy expenditure and improves insulin sensitivity in aged mice, revealing the important role of GHS-R in metabolism [13]. We also found ghrelin and GHS-R to be involved in the development of obesity by regulating thermogenic activation of adipose tissue and energy dissipation [13, 14]. *Ghsr*-null mice are cold-resistant and have enhanced thermogenesis [13], the thermogenic effect of GHS-R could be centrally neuron-mediated and/or peripherally BAT-mediated. We have evidence that *Ghsr* gene knockdown in brown adipocytes activates thermogenic signaling [14], suggesting that the GHS-R may directly regulate thermogenesis in BAT.

The present study aims to define the cell-autonomous effect of GHS-R in BAT under homeostasis and obese metabolic conditions. To tissue-specifically delete GHS-R in BAT, we used the Cre-*lox*P system by breeding Cre mice driven by brown adipocyte-specific UCP1 promoter [15] with flox mice flanking GHS-R we previously reported [16]. The thermogenic and mitochondrial gene expression profile was studied under both homeostatic and obese states, under normal or cold temperature.

## Materials and methods

### Animals

All experimental procedures were approved by the Institutional Animal Care and Use Committee (IACUC) at Baylor College of Medicine. BAT-specific *Ghsr* knockout mice were generated by breeding the GHS-R floxed mice [16] with *UCP1*-Cre^ER^ mice that carry a tamoxifen-inducible Cre recombinase generated by the co-author Christian Wolfrum‘s group, which has been shown to specifically target brown adipocytes in BAT and beige cells in subcutaneous fat [15]. The male *Ghsr*^*f/f*^ (WT) and *UCP1*-Cre^ER^/*Ghsr*^*f/f*^ mice were induced at 8-weeks of age by orally gavaging tamoxifen (dissolved in peanut oil) for 5 days as previously described [15]. Mice were housed in the animal facility of Baylor College of Medicine, maintained at standard conditions with 12 h light/dark cycles with free access to water and food. In the current study, age-matched male *Ghsr*^*f/f*^ (WT) and *UCP1*-Cre^ER^/*Ghsr*^*f/f*^ mice (n = 6 per genotype) were fed with either regular diet (RD) or high-fat diet (HFD) with 42% fat, 42.7% carbohydrates, and 15.2% protein in calories (TD. 88137 Western diet, Harlan Teklad, Madison, WI). At the termination, mice were sacrificed with CO_2_ inhalation and followed by cervical dislocation, according to the protocol approved by the IACUC. BAT was dissected from the interscapular region, instantly frozen in N2, then stored in -80°C until use.

### Body composition and body temperature

Body composition and body temperature data were obtained as we previously described [16]. Whole-body composition was monitored using Echo MRI-100 whole-body composition analyzer (Echo Medical Systems, Houston, TX). For the cold exposure study, mice were individually caged in 4°C cold room for 4‒6 h, with free access to food and water. Core body temperature was measured with a rectal probe attached to a TH-8 Thermalert monitoring thermometer (Physitemp Instruments Inc. Clifton, NJ). Body temperature was assessed hourly for the duration of the experiment. For humane consideration required by IACUC policy, the experiment was aborted when a temperature lower than 25°C detected in the mice.

### RT-qPCR analysis

Total RNA was isolated using TRIzol® Reagent (Invitrogen, Carlsbad, CA) or RNeasy Mini kit (QIAGEN). RNA samples were treated with RNase-free DNase (Ambion, Austin, TX). Reverse transcription was performed with Superscript III First Strand Synthesis System (Invitrogen, Carlsbad, CA). Quantitative real-time PCR was performed using SYBR Green Supermix (Bio-Rad Lab., Hercules, CA). Relative gene expression levels were normalized using *18S* rRNA. The primer sequences are listed in S1 Table.

### Statistical analysis

All data were expressed as the mean ± SEM. Differences were analyzed by one-way ANOVA followed by Tukey’s post-hoc multiple comparison tests, as appropriate. An unpaired 2-tailed *t*-test was used for analysis within two groups. Significance was determined as *p* < 0.05.

## Results

### BAT-specific *Ghsr* gene knockout validation

**Fig 1:**
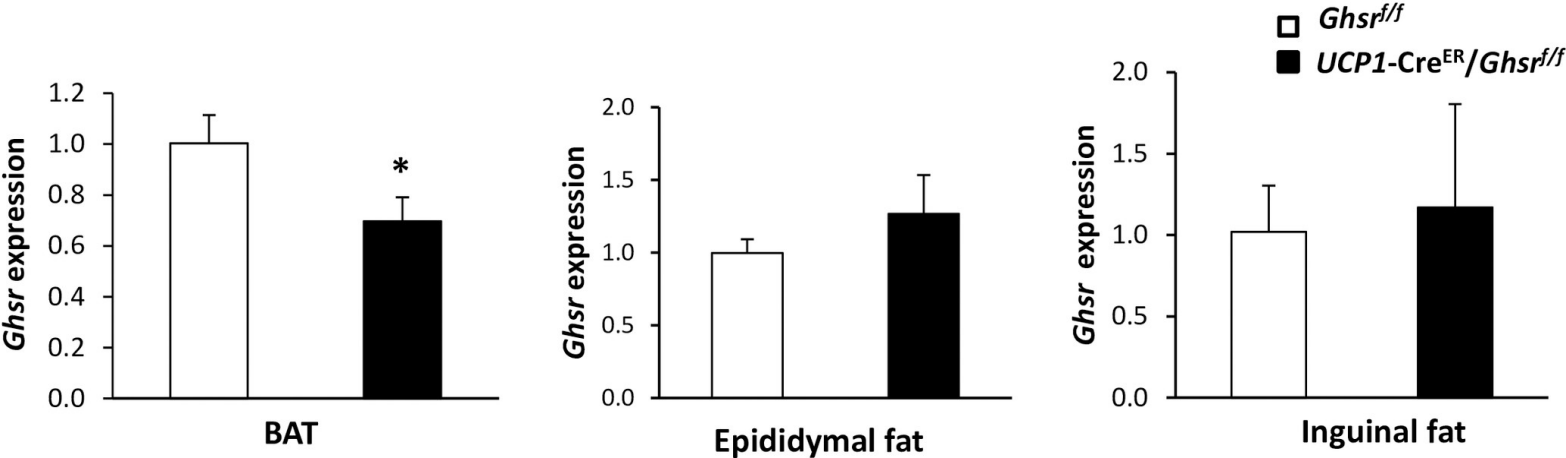
Validation of *Ghsr* expression in *UCP1*-Cre^ER^/*Ghsr*^*f/f*^ and control mice. *Ghsr* mRNA expression levels in BAT, epididymal fat, and inguinal fat. All data were expressed as the mean ± SEM. n = 4‒5, *P < 0.05, *Ghsr*^*f/f*^ *vs*. *UCP1*-Cre^ER^/*Ghsr*^*f/f*^.

To selectively disrupt *Ghsr* expression in BAT, the mice with *Ghsr* floxed allele (*Ghsr*^*f/f*^) were bred with *UCP1*-Cre^ER^ mice to excise the GHS-R open reading frame in a BAT-specific manner to generate *UCP1*-Cre^ER^/*Ghsr*^*f/f*^ mice. GHS-R deletion was induced by oral gavage of tamoxifen at 8-weeks of age, and the expression of *Ghsr* was determined in different fat depots at least 1 month after the induction. The *UCP1*-Cre^ER^/*Ghsr*^*f/f*^ mice showed reduced expression of *Ghsr* in BAT, but not in epididymal or inguinal fat tissues; this suggests that *Ghsr* gene deletion is restricted to BAT fat depots (**Fig 1**). The expression levels indicated that there is about 40% reduction of *Ghsr* in BAT of *UCP1*-Cre^ER^/*Ghsr*^*f/f*^ mice compared with that of *Ghsr*^*f/f*^ mice. That is known that brown adipocytes are only counted for 20–50% of the total cell content of BAT; stromal vascular fraction (SVF), a heterogeneous cell faction composites of immune cells, endothelial cells and fibroblasts, is also present in BAT [15, 17, 18]. Thys, the 40% reduced expression of GHS-R in BAT in *UCP1*-Cre^ER^/*Ghsr*^*f/f*^ mice likely reflect a near complete deletion of GHS-R in brown adipocytes.

### Body composition in RD-fed mice

**Fig 2:**
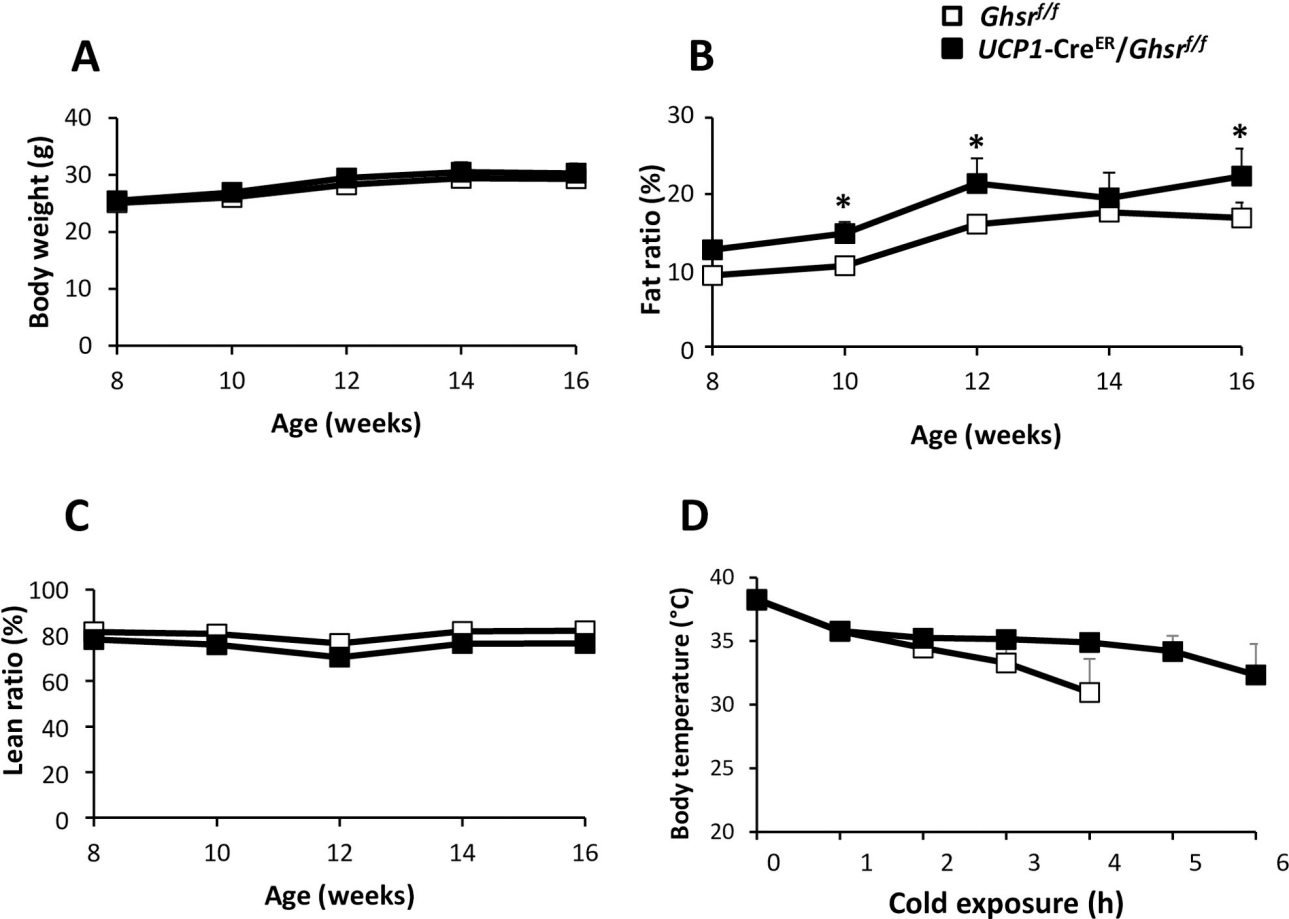
Body weight and body composition of RD-fed mice. (**A**) Body weight changes, (**B**) fat percentage, (**C**) lean percentage of *Ghsr*^*f/f*^ mice and *UCP1*-Cre^ER^/*Ghsr*^*f/f*^ mice. (**D**) Rectal temperatures of *Ghsr*^*f/f*^ and *UCP1*-Cre^ER^/*Ghsr*^*f/f*^ mice. 20-week-old mice were individually caged in a 4°C cold room for 6 h, with free access to food and water. The rectal temperatures were collected every hour. All data were expressed as the mean ± SEM. n = 3‒6, *P < 0.05, *Ghsr*^*f/f*^ *vs*. *UCP1*-Cre^ER^/*Ghsr*^*f/f*^.

The body weights of *Ghsr*^*f/f*^ and *UCP1*-Cre^ER^/*Ghsr*^*f/f*^ mice were no difference for up to 16-weeks of age (**Fig 2A**). Intriguingly, the fat percentage was increased in *UCP1*-Cre^ER^/*Ghsr*^*f/f*^ mice, while the relative changes of the lean mass remained insignificant between the two genotypes (**Fig 2B and 2C**). To evaluate the thermogenic response to cold stress, the mice were placed in 4°C for 4‒6 h and rectal temperature was measured every hour. The body temperature was not different between the two genotypes at 0 h and 1 h. However, *UCP1*-Cre^ER^/*Ghsr*^*f/f*^ mice showed mild resistance to cold stress after 2 h time point; the difference of the two genotypes diverged and became more pronounced with time (**Fig 2D**). After 4 h, the body temperatures of many *Ghsr*^*f/f*^ mice was lower than 25°C, and we had to abort the experiment (per IACUC requirement), while the body temperatures of all *UCP1*-Cre^ER^/*Ghsr*^*f/f*^ mice maintained above 35°C (**Fig 2D**).

### Thermogenic gene expression profile in BAT of RD-fed mice

**Fig 3:**
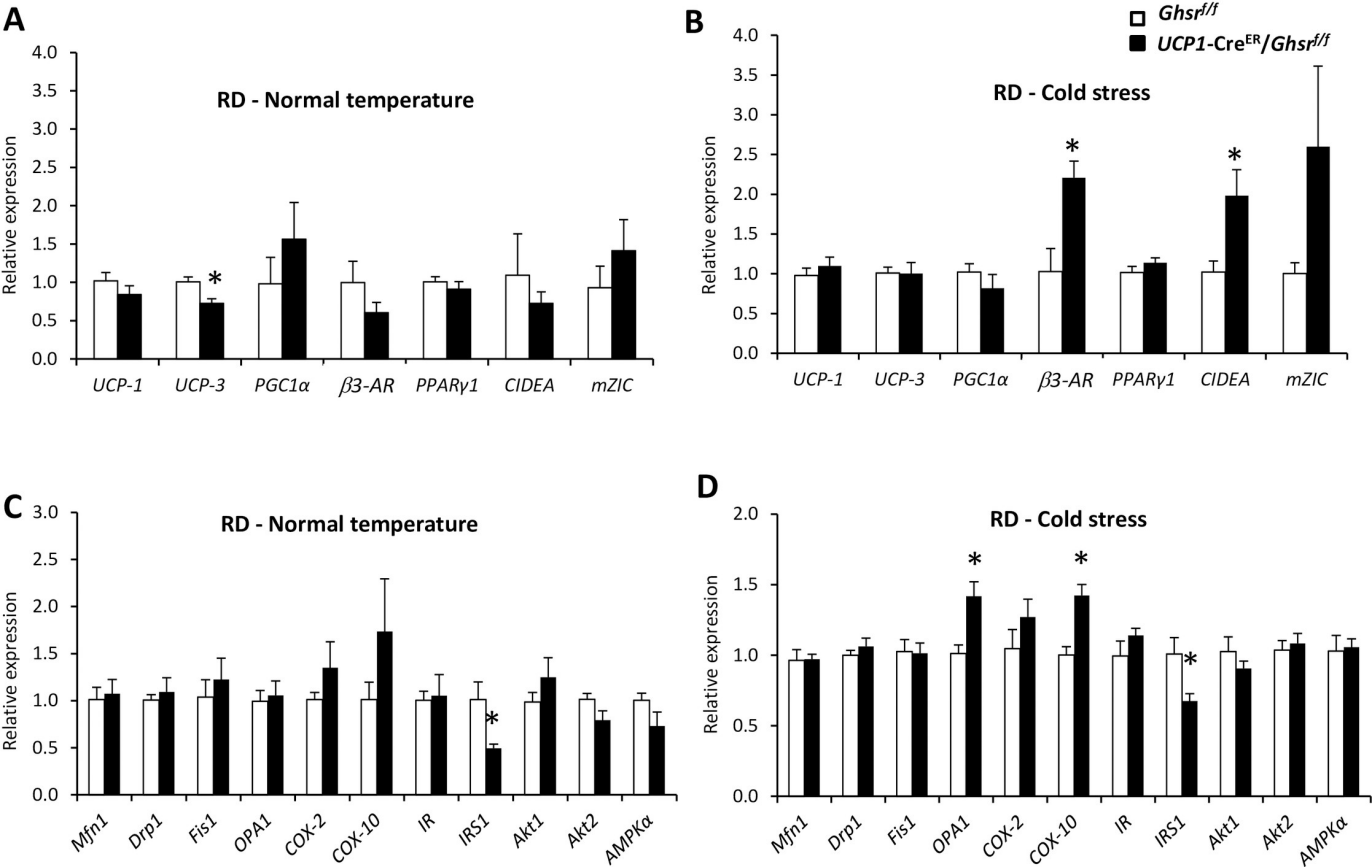
Gene expression profile of BAT in RD-fed mice. BAT from 20-week old male RD fed *Ghsr*^*f/f*^ and *UCP1*-Cre^ER^/*Ghsr*^*f/f*^ mice were collected either under normal housing temperature or after 6 h of 4°C cold exposure. (**A**) Thermogenic-related gene mRNA expression levels in the BAT under normal housing temperature. (**B**) Thermogenic-related gene mRNA expression levels in the BAT under 4°C cold exposure. (**C**) Mitochondrial and insulin signaling related gene mRNA expression levels in the BAT under normal housing temperature. (**D**) Mitochondrial and insulin signaling related gene mRNA expression levels in the BAT under 4°C cold exposure. All data were expressed as the mean ± SEM. n = 3‒6, *P < 0.05, *Ghsr*^*f/f*^ *vs*. *UCP1*-Cre^ER^/*Ghsr*^*f/f*^.

Next, we analyzed the thermogenic gene expression profile. Under normal temperature, the mitochondrial gene UCP3 expression was significantly decreased in *UCP1*-Cre^ER^/*Ghsr*^*f/f*^ mice compared with that of *Ghsr*^*f/f*^ mice; no pronounced changes were observed in other thermogenic genes (**Fig 3A**). When mice were exposure to 4°C cold for 6 h, the expression of β3-adrenergic receptor (β3-AR) and brown-fat marker cell death-induced DFFA-like effector A (CIDEA) [19] showed significant increases in the BAT of *UCP1*-Cre^ER^/*Ghsr*^*f/f*^ mice (**Fig 3B**). These increases suggest an enhanced thermogenic activation in BAT of the *UCP1*-Cre^ER^/*Ghsr*^*f/f*^ mice under cold stress. In line with the temperature phenotype, the expression of mitochondrial biogenesis genes including optic atrophy-1 (OPA-1) and cytochrome c oxidase-10 (Cox-10), in BAT of *UCP1*-Cre^ER^/*Ghsr*^*f/f*^ mice was not changed under normal temperature (**Fig 3C**) but increased under cold stress (**Fig 3D**). Intriguingly, insulin receptor substrate 1 (IRS1) expression was suppressed in BAT of *UCP1*-Cre^ER^/*Ghsr*^*f/f*^ mice, under both normal and cold temperatures.

### Body composition in HFD-fed mice

**Fig 4:**
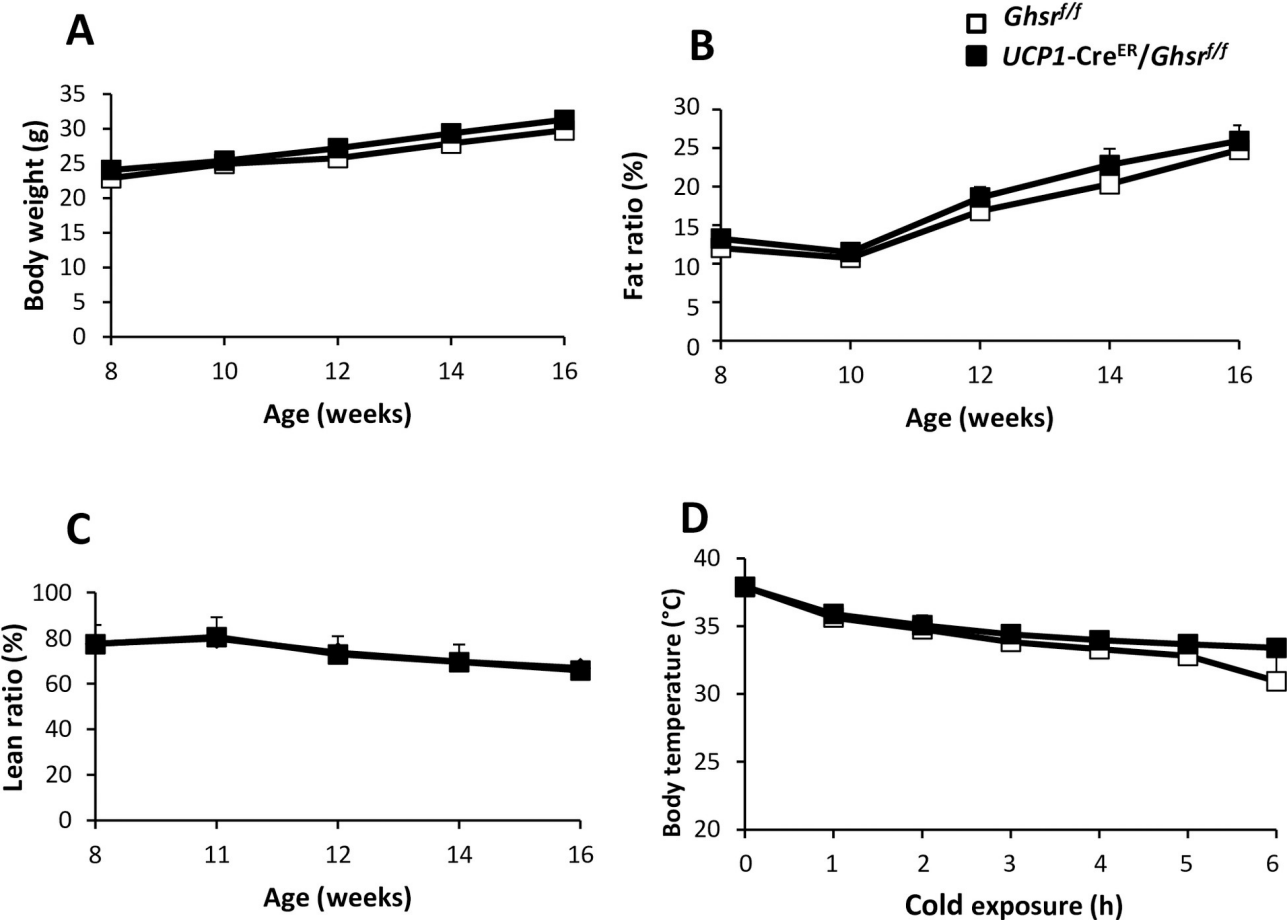
Body weight and body composition of HFD-fed mice. 20-week old male *Ghsr*^*f/f*^ and *UCP1*-Cre^ER^/*Ghsr*^*f/f*^ mice fed HFD. (**A**) Body weight changes, (**B**) fat percentage, (**C**) lean percentage of *Ghsr*^*f/f*^ mice and *UCP1*-Cre^ER^/*Ghsr*^*f/f*^ mice. (**D**) Rectal temperature of mice individually caged in 4°C cold room for 6 h, with free access to food and water. The rectal temperatures were collected every hour. All data were expressed as the mean ± SEM. n = 3‒6, *P < 0.05, *Ghsr*^*f/f*^ *vs*. *UCP1*-Cre^ER^/*Ghsr*^*f/f*^.

To further assess whether GHS-R in BAT affects diet-induced thermogenesis, the mice were fed with HFD from 8-weeks of age for the period of 8 weeks. The mice of both genotypes gained more body weight and fat content under HFD. The body weights (**Fig 4A**), fat percentages (**Fig 4B**), and lean percentages (**Fig 4C**) between the *UCP1*-Cre^ER^/*Ghsr*^*f/f*^ and *Ghsr*^*f/f*^ mice were similar. The mice were subjected to cold stress and body temperature was monitored hourly. Contrary to the RD-fed mice, no differences were detected in core body temperatures between *UCP1*-Cre^ER^/*Ghsr*^*f/f*^ and control mice (**Fig 4D**). It is interesting to note the HFD-fed control mice were able to maintain a higher temperature than RD-fed control mice under cold exposure. For example, at 4 h time point, body temperature of HFD-fed control mice maintained at ~34°C (**Fig 4D**), while the temperature of most RD-fed control mice dropped below 25°C (the experiment had to be aborted) and the few remaining RD-fed mice kept temperature at ~31°C (**Fig 2D**).

### Thermogenic gene expression profile in BAT of HFD-fed mice

**Fig 5:**
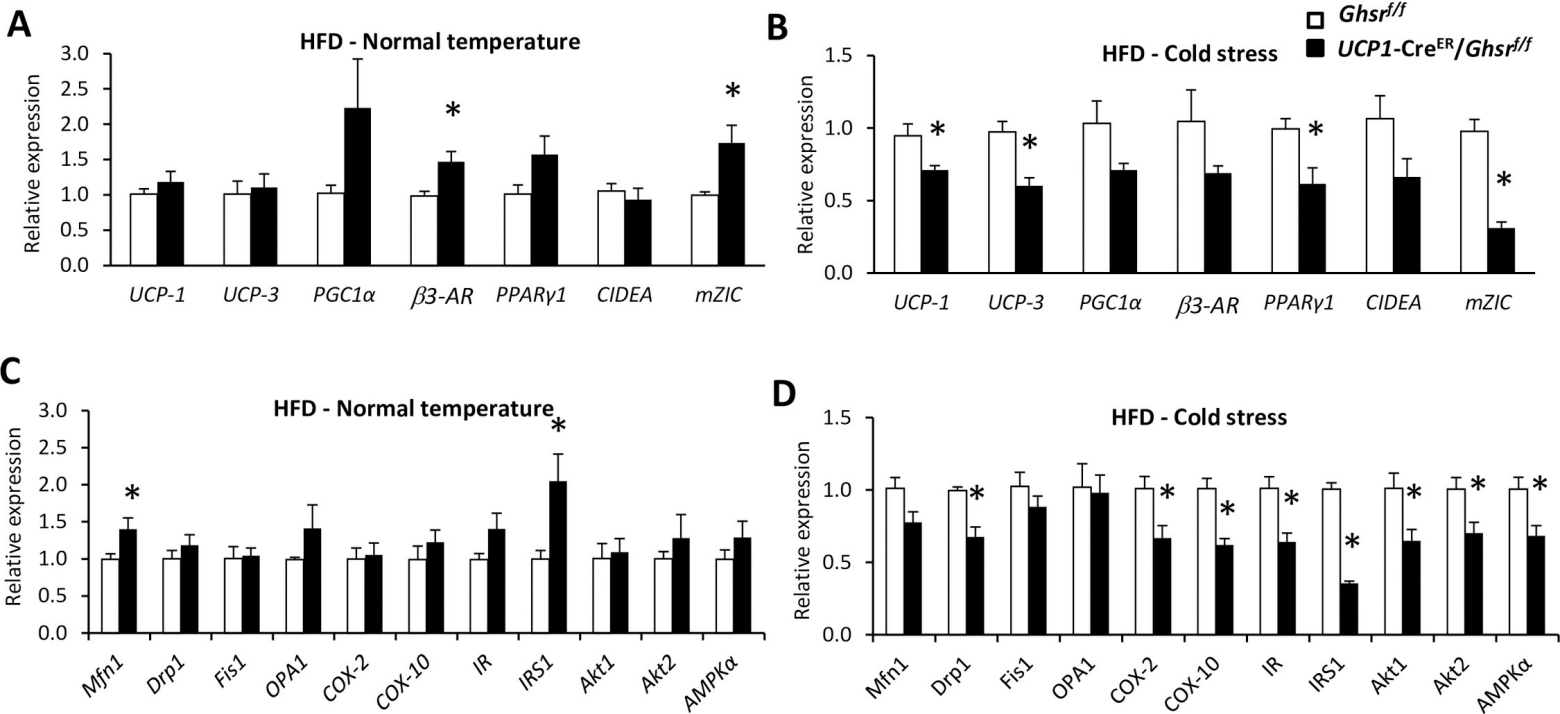
Gene expression profile of BAT in mice fed HFD. BAT from 20-week old male HFD-fed *Ghsr*^*f/f*^ and *UCP1*-Cre^ER^/*Ghsr*^*f/f*^ mice were either collected under normal housing temperature or after 6 h of 4°C cold exposure. (**A**) Thermogenic-related *gene* expression under normal housing temperature. (**B**) Thermogenic-related *gene* expression under 4°C cold exposure. (**C**) Expression of mitochondrial and insulin signaling-related genes under normal housing temperature. (**D**) Expression of mitochondrial and insulin signaling-related genes under 4°C cold exposure. All data were expressed as the mean ± SEM. n = 3‒6, *P < 0.05, *Ghsr*^*f/f*^ *vs*. *UCP1*-Cre^ER^/*Ghsr*^*f/f*^.

Under normal temperature, the expression of β3-AR and brown-fat marker Zinc finger protein 1 (ZIC1) was significantly increased in HFD-fed *UCP1*-Cre^ER^/*Ghsr*^*f/f*^ in comparison to that of *Ghsr*^*f/f*^ mice (**Fig 5A**). The expression pattern was similar to that of RD-fed *UCP1*-Cre^ER^/*Ghsr*^*f/f*^ mice exposed to cold. Surprisingly, this effect was reversed when mice were challenged with cold stress, showing reduced expression of UCP1, UCP3, PPARγ1, and ZIC1 in BAT of HFD-fed *UCP1*-Cre^ER^/*Ghsr*^*f/f*^ mice (**Fig 5B**). Consistently, the expression of mitochondrial dynamic genes of Mitofusin-1 (Mfn-1) and IRS1 was increased in BAT of *UCP1*-Cre^ER^/*Ghsr*^*f/f*^ mice under normal temperature (**Fig 5C**), but the expression of mitochondrial dynamics and biogenesis (Drp1, Cox2, Cox10), insulin signaling (IR, IRS1, Akt1, Akt2), and master metabolic regulator AMPKα genes was downregulated in BAT of HFD-fed *UCP1*-Cre^ER^/*Ghsr*^*f/f*^ under cold stress (**Fig 5D**).

## Discussion

Characterizing the role of GHS-R in BAT thermoregulation is the primary focus of this study. To investigate the cell-autonomous effect of GHS-R in brown adipocytes, we generated brown adipocyte-specific *Ghsr* knockout mice. The *UCP1* gene promoter-driven Cre mice have been used in BAT-specific transgenic mouse studies for both gene knockout and knockin, which has demonstrated good specificity in targeting brown adipocytes [15, 20]. Our validation data showed that the efficiency of the *Ghsr* deletion in BAT was about 40% in *UCP1*-Cre^ER^/*Ghsr*^*f/f*^ mice. Brown adipocytes are known only counted for 20‒50% of total cell population of BAT, while the rest cells are mostly made of SVF that includes immune cells such as macrophages [15, 17, 18, 21]. 40% reduced GHS-R expression in BAT of *UCP1*-Cre^ER^/*Ghsr*^*f/f*^ mice may be exclusively contributed by gene deletion in brown adipocytes, and GHS-R deletion in brown adipocytes likely achieved a remarkable near 100% efficiency. We have showed that GHS-R is expressed in peritoneal macrophages, adipose tissue macrophages and macrophages cell line [13, 22]. The remaining intact GHS-R expression in BAT of *UCP1*-Cre^ER^/*Ghsr*^*f/f*^ mice was likely contributed by immune cells such as macrophages in the SVF fraction. The *UCP1*-Cre^ER^/*Ghsr*^*f/f*^ mice showed thermogenic activation in BAT and higher body temperatures under cold stress. β3-AR is known to control brown adipose UCP1 tone and cold response [23, 24]. Consistently, *UCP1*-Cre^ER^/*Ghsr*^*f/f*^ mice showed an increase of β3-AR expression in BAT with RD-feeding under cold stress, and with HFD-feeding under normal housing temperature. These results support that GHS-R has cell-autonomous effects in BAT, and it directly regulates the thermogenic activity in BAT.

We found that the thermogenic gene profiles in BAT from *UCP1*-Cre^ER^/*Ghsr*^*f/f*^ mice were primarily pronounced under cold but not under normal housing temperature, similar to our observation in global *Ghsr* knockout mice [13]. It has been reported that ghrelin suppresses thermogenic action, and the effect is more pronounced under obesity [16, 25]. Our data showed that HFD enhances cold-resistance more than RD-feeding in both *Ghsr*^*f/f*^ and *UCP1*-Cre^ER^/*Ghsr*^*f/f*^ mice. Interestingly, *UCP1*-Cre^ER^/*Ghsr*^*f/f*^ mice showed the thermo-protective effect of cold under RD but not under HFD, suggesting the effect of GHS-R in BAT thermogenesis is influenced by metabolic state. More intriguingly, under HFD-feeding, the thermogenic gene expression profile of BAT from *UCP1*-Cre^ER^/*Ghsr*^*f/f*^ mice were opposite under normal and cold temperature. These intriguing data suggest that the GHS-R signal in BAT senses the metabolic changes of the microenvironment and differentially regulates thermogenic activity in a thermo- and metabolic state-dependent manner. This new findings have following implications: 1) GHS-R signaling in BAT contributes to diet-induced obesity by suppressing thermogenesis; 2) The thermo-beneficial effect of GHS-R deficiency in BAT is diminished in the obese animals, suggesting that GHS-R mediated thermogenic signaling is impaired in obesity; 3) GHS-R antagonists likely activate thermogenesis under normal metabolic state but suppress thermogenesis under obese state, this differential effect must be taken into consideration in assessment of the therapeutic potential of GHS-R antagonism.

That is known that food intake can potentially contribute to or compensate for the thermogenic phenotype. Our previous study of *ap2*-Cre/*Ghsr*^*f/f*^ mice (with GHS-R deletion BAT) showed there is no difference in food intake [32]. That is likely other factors contribute to the differential outcomes of *UCP1*-Cre^ER^/*Ghsr*^*f/f*^ mice under different metabolic conditions. We previously reported that GHS-R ablation increased BAT thermogenesis by activating central sympathetic nervous system (SNS) mediated norepinephrine-induced β3-AR in BAT of aged mice, suggesting that different metabolic/physiological conditions such as aging and obesity may affect thermogenesis via both central and peripheral mechanisms [14]. Some studies suggest that cold stress combined with HFD can trigger the release of neuropeptide Y (NPY) from sympatric neurons into adipose tissue, in turn upregulates NPY and its Y2 receptors (NPY2R) in a glucocorticoid-dependent manner in the abdominal fat, leading to increased fat content and obesity [26]. This might explain in some extent our results in HFD-fed mice under cold exposure, where HFD stimulates the synaptic nervous activity and cold exposure further increases stress response, thereby boosting thermogenic activation in BAT. The thermogenic phenotype of *UCP1*-Cre^ER^/*Ghsr*^*f/f*^ mice may be partially determined by direct cell-autonomous effects of GHS-R in BAT and partially determined by indirect central feedback regulation.

Circulating ghrelin levels are usually low after feeding and in obesity [27]. We have showed that GHS-R expression is increased by under HFD and aging in tissues such as brain and BAT [14, 16]. The different expression levels of GHS-R in BAT or circulating ghrelin levels may affect the outcome of GHS-R suppression in BAT, resulting in different outcomes between RD and HFD mice, as well as by cold stress. However, ghrelin is primarily produced by enteroendocrine cells in stomach and released into circulation [28]. The enteroendocrine cells in the stomach are not a UCP1-cre target, thus we don’t expect a difference in ghrelin levels between control and brown fat GHS-R knockout mice under a given dietary or temperature condition. To ultimately confirm he phenotype of brown fat-specific GHSR deleted mice is not affected by endogenous ghrelin, it is possible to study the thermogenic phenotype of *UCP1-cre;Ghsr*^*f/f*^ mice under ghrelin deficiency background.

Our previous studies showed that global *Ghsr* deletion not only reduces adiposity but also improves insulin sensitivity by inducing thermogenesis, suggesting that the suppression of GHS-R signaling activates thermogenic signaling in BAT to promote heat production, thus increasing energy expenditure [13, 14]. There is evidence suggesting that central temperature sensing and signaling-driven thermogenesis are enhanced by local and endocrine signals surrounding BAT [29, 30]. Due to the limitation of global gene deletion, it is difficult to decipher the site(s) of GHS-R mediated thermoregulation was central or peripheral. Our neuronal *Ghsr* knockout mice showed the most robust thermogenic phenotype and increased SNS-induced norepinephrine release, suggesting that GHS-R mediated thermogenesis is predominately centrally regulated [14, 16, 31]. We previously reported increased thermogenesis and UCP1 expression in BAT of *ap2*-Cre/*Ghsr*^*f/f*^ mice, where the knockdown effect of GHS-R was not only taking place in adipose tissues, but also partially in hypothalamus [32]. The current study using a brown adipocyte-specific Cre driver enables us to truly assess the BAT-specific role of GHS-R in thermogenesis. However, the thermo-activation in *UCP1*-Cre^ER^/*Ghsr*^*f/f*^ mice was much milder than in that of global and neuronal knockout mice; this suggests that while GHS-R directly regulates thermogenic signaling in BAT, its overall *in vivo* impact is minimal. Therefore, it is conceivable that GHS-R mediated central circuits have a dominant effect on thermogenesis than the direct thermoregulation of GHS-R in brown adipocytes.

## Conclusions

The results of this study indicate that GHS-R cell-autonomously regulates thermogenic signaling in BAT. Under a normal metabolic state of RD feeding, GHS-R suppression in BAT activates thermogenesis under cold but not under normal temperature. Under obese state, GHS-R suppression in BAT activates thermogenesis under normal temperature but inhibits thermogenesis under cold exposure. While our data support that GHS-R suppression-induced thermogenic activation in BAT may have therapeutic potential, its application is likely to be limited because the direct effect of GHS-R on thermoregulation in BAT requires cold stimulation, and it only appears to be effective under a normal metabolic state but not the obese state. Overall, the results support that nutrient-sensing GHS-R, acting as a “metabolic thermostat” in BAT, modulates thermoregulation in BAT in a metabolic state-dependent and temperature-dependent manner. GHS-R antagonists may have therapeutic potential for weight control, but their application is likely limited to individuals with a normal metabolic state rather than obese subjects.

## Supporting information

S1 Fig Weight of different fat deports of HFD-fed mice. 20-week old male *Ghsr*^*f/f*^ and *UCP1*-Cre^ER^/*Ghsr*^*f/f*^ mice fed HFD. The weights of epididymal fat, inguinal fat and BAT at termination of the experiment. All data were expressed as the mean ± SEM. n = 3‒6, *P < 0.05, *Ghsr*^*f/f*^ *vs*. *UCP1*-Cre^ER^/*Ghsr*^*f/f*^.
(PPTX)

S1 Table RT-qPCR primers. (DOCX)

S1 Dataset (XLSX)
