# Supplementary material for: GHS-R in brown fat potentiates differential thermogenic responses under metabolic and thermal stresses
Source: PLoS One. 2021 Apr 1;16(4):e0249420. doi: 10.1371/journal.pone.0249420 (PMC8016305; doi:10.1371/journal.pone.0249420)
Supplement: S1 Fig — 20-week old male Ghsrf/f and UCP1-CreER/Ghsrf/f mice fed HFD. The weights of epididymal fat, inguinal fat and BAT at termination of the experiment. All data were expressed as the mean ± SEM. n = 3‒6, *P < 0.05, Ghsrf/f vs. UCP1-CreER/Ghsrf/f. (PPTX) [file pone.0249420.s001.pptx]

## Slide 1
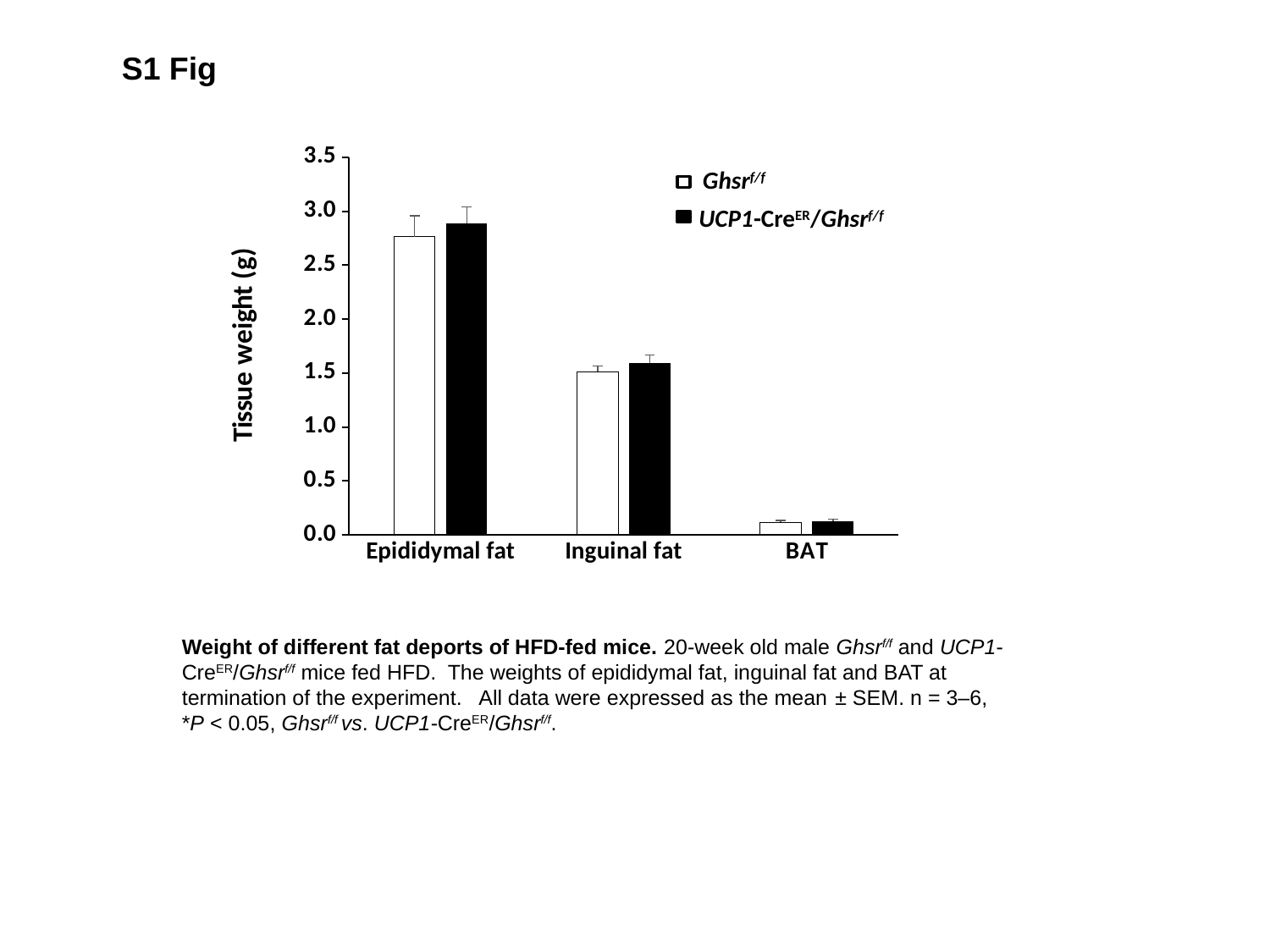

S1 Fig
### Chart
| Category | | |
|---|---|---|
| Epididymal fat | 2.765283333333333 | 2.88865 |
| Inguinal fat | 1.5143833333333332 | 1.5935833333333331 |
| BAT | 0.11493333333333333 | 0.125 |Ghsrf/f
UCP1-CreER/Ghsrf/f
Weight of different fat deports of HFD-fed mice. 20-week old male Ghsrf/f and UCP1-CreER/Ghsrf/f mice fed HFD. The weights of epididymal fat, inguinal fat and BAT at termination of the experiment. All data were expressed as the mean ± SEM. n = 3‒6, *P < 0.05, Ghsrf/f vs. UCP1-CreER/Ghsrf/f.
