# Supplementary material for: GHS-R in brown fat potentiates differential thermogenic responses under metabolic and thermal stresses
Source: PLoS One. 2021 Apr 1;16(4):e0249420. doi: 10.1371/journal.pone.0249420 (PMC8016305; doi:10.1371/journal.pone.0249420)
Supplement: S1 Table — (DOCX) [file pone.0249420.s002.docx]

**S1 Table. RT-qPCR primers**

| **Genes** | **Primer Sequence (5’ to 3’)** |
| --- | --- |
| GHSR-1a | GGACCAGAACCACAAACAGACA |
|  | CAGCAGAGGATGAAAGCAAACA |
| UCP-1 | GTGAAGGTCAGAATGCAAGC |
|  | AGGGCCCCCTTCATGAGGTC |
| UCP-3 | GAGCGGACCACTCCAGCGTC |
|  | TGAGACTCCAGCAACTTCTC |
| PGC1a | TTTTTGGTGAAATTGAGGAATGC |
|  | CGGTAGGTGATGAAACCATAGCT |
| β3-AR | TGCCAACTCTGCCTTCAACCCGCTC |
|  | CGCTCACCTTCATAGCCATCAAACC |
| PPARγ1 | cacgttctgacaggactgtgt |
|  | cagcaaccattgggtcagctc |
| CIDEA | CCATCCCTCTGCATGGAGTACCTTTC |
|  | CCTCCTGTGTCACATGGAACCAG |
| Zic1 | GCGTTCAGAGAACCTCAAGATCCAC |
|  | AAAGGTAGGGCTTGTCGCTCGTG |
| Mfn1 | TCTCCAAGCCCAACATCTTCA |
|  | ACTCCGGCTCCGAAGCA |
| Drp1 | CGGTTCCCTAAACTTCACGA |
|  | GCACCATTTCATTGTCACG |
| Fis1 | CCGGCTCAAGGAATATGAAA |
|  | CCATGCCTACCAGTCCATCT |
| OPA1 | GATGACACGCTCTCCAGTGAAG |
|  | CTCGGGGCTAACAGTACAACC |
| COX2 | AACCATAGGGCACCAATGATAC |
|  | GGATGGCATCAGTTTTAAGTCC |
| COX10 | AGCAGGAAGCGTATTTTGGT |
|  | GTTTGGGAAGCAGTTTGGAT |
| IR | CAAAAGCACAATCAGAGTGAGTATGAC |
|  | ACCACGTTGTGCAGGTAATCC |
| IRS1 | GCCTGGAGTATTATGAGAACGAGAA |
|  | GGGGATCGAGCGTTTGG |
| Akt1 | GACCCACGACCGCCTCTG |
|  | GACACAATCTCCGCACCATAGAAG |
| Akt2 | GAGGACGCCATGGATTACAAG |
|  | GACAGCTACCTCCATCATCTCAGA |
| AMPKa2 | CAGGCCATAAAGTGGCAGTTA |
|  | AAAAGTCTGTCGGAGTGCTGA |
